# Increased Risk of Colorectal Cancer in Type 2 Diabetes Is Independent of Diet Quality

**DOI:** 10.1371/journal.pone.0074616

**Published:** 2013-09-12

**Authors:** Soghra Jarvandi, Nicholas O. Davidson, Mario Schootman

**Affiliations:** 1 Division of Health Behavior Research, Department of Medicine, Washington University School of Medicine, Saint Louis, Missouri, United States of America; 2 Division of Gastroenterology, Department of Medicine, Washington University School of Medicine, Saint Louis, Missouri, United States of America; 3 Alvin J. Siteman Cancer Center at Barnes-Jewish Hospital and Washington University School of Medicine, Saint Louis, Missouri, United States of America; Robert Gordon University, United Kingdom

## Abstract

**Background:**

Poor diet increases the risk of both colorectal cancer and type 2 diabetes. We investigated the role of diet in the association between diabetes and colorectal cancer.

**Methods:**

We analyzed data from 484,020 individuals, aged 50–71 years who participated in the prospective National Institutes of Health-AARP Diet and Health Study and were cancer free at baseline (1995–1996). History of diabetes was self-reported. Diet quality was measured with the Healthy Eating Index-2005 (HEI-2005), using a self-administered food-frequency questionnaire. Cox regression models were constructed to estimate the hazard ratios (HR) and 95% confidence intervals (CI) of first primary incident colorectal cancer, overall and by anatomical location.

**Results:**

During an average follow-up of 9.2 years, we identified 7,598 new cases of colorectal cancer. After controlling for non-dietary confounders, diabetes was associated with increased risk of colorectal cancer (HR 1.27, 95% CI: 1.18, 1.36). Further adjustment for diet quality did not attenuate this association. Diabetes was associated with a HR of 1.23 (95% CI: 1.07, 1.40) in individuals with good diet (quartile 4 of HEI-2005) and 1.58 (95% CI: 1.34, 1.86) in those with poor diet (quartile 1 of HEI-2005), compared to those with no diabetes and good diet. Moreover, diabetes was associated with a stronger risk of proximal than distal colon cancer (HR: 1.33 vs. HR: 1.20), while poor diet was associated with a weaker risk of proximal colon cancer (HR: 1.18 vs. HR: 1.46).

**Conclusion:**

Diabetes and poor diet, independently and additively are associated with the increased risk of colorectal cancer.

## Introduction

Type 2 diabetes is a common chronic disease with a dramatic escalating prevalence worldwide. In the United States, prevalence of diabetes doubled from 4% in 2000 [1] to 8% in 2010 [2]. People with type 2 diabetes are at increased risk of colorectal cancer compared with individuals without diabetes [3], [4]. However, a key question is whether this increased risk is merely because of the shared risk factors, such as poor diet, low level of physical activity and obesity, or whether other diabetes-related factors increase the risk of colorectal cancer.

A relatively poor dietary intake may be in part responsible for the increased risk of colorectal cancer among people with type 2 diabetes; a typical Western dietary pattern has been associated with the increased risk of both colorectal cancer [5] and type 2 diabetes [6]. However, the role of dietary factors in the association between diabetes and colorectal cancer has not been examined in sufficient detail. A recent systematic review [7] indicates that most of the previous studies, while adjusted for physical activity and obesity, either did not control for specific dietary factors or just included energy and/or only a few selected food items. Only two cohort studies, one in both men and women [4] and the other in men only [8], included a comprehensive list of dietary variables including fruit and vegetables and either cereals, meat and milk [4] or dairy and red meat [8]. However, no studies have gone beyond the assessment of selected dietary factors to focus on the entire diet, i.e. diet quality, in the association between diabetes and colorectal cancer. Studies generally suggest that the association between overall diet composition and colorectal cancer is more consistent than that of individual foods or nutrients [9], . Diet quality examines diet as a whole rather than individual food items or nutrients, and thus integrates the real-world complexity of food intake and the interactions between dietary constituents [11]. In addition, the diet quality approach facilitates examination of the combined effects of poor diet and diabetes on the risk of colorectal cancer, an important public health issue that has not yet been studied.

Diabetes and diet quality may play different roles in the development of proximal or distal colon because of different pathogenicity mechanisms and inherent differences between these anatomical sub-sites [12]. Previous work on the NIH-AARP population reported an inverse association between diet quality and the risk of distal colon cancer [10]. However, there is conflicting evidence regarding the site-specific association (i.e. proximal versus distal) between colon cancer and diabetes [3], [8], [13], [14]. Such associations, if any, might illuminate potential etiological distinctions between diet- and diabetes-related colorectal cancer.

Our goal was to investigate the role of diet quality in the association between diabetes and colorectal cancer. The specific objectives were to: 1) examine the association between diabetes and risk of colorectal cancer and test whether diet quality confounds this association; 2) estimate the combined effect of diabetes and poor diet on the risk of colorectal cancer; and 3) examine the site-specific association between colorectal cancer and diabetes and compare the result with that of the colorectal cancer and diet quality.

## Methods

### Ethics Statement

The NIH-AARP Diet and Health Study was approved by the Special Studies Institutional Review Board of the National Cancer Institute.

We used data from the National Institute of Health-AARP (NIH-AARP) Diet and Health Study. Details of the NIH-AARP Diet and Health Study procedures and design have been reported previously [15]. Briefly, in 1995–1996 a baseline questionnaire was mailed to 3.5 million AARP members aged 50–71 living in 6 US states (California, Florida, Louisiana, New Jersey, North Carolina, and Pennsylvania) and 2 metropolitan areas (Atlanta, Georgia, and Detroit, Michigan). The questionnaire, which included demographic, dietary and lifestyle data, was returned by 617,119 persons. After excluding unsatisfactory questionnaires, the baseline cohort includes 566,399 respondents. In 1996–1997, a second questionnaire (risk factor questionnaire) was mailed to people who did not report a cancer history of one of three cancer types including breast, prostate and colorectal at baseline questionnaire. The risk factor questionnaire was completed by 334,908 individuals (response rate: 63%). The NIH-AARP Diet and Health Study was approved by the Special Studies Institutional Review Board of the US National Cancer Institute (NCI).

### Study Population

Of the 566,399 baseline respondents, we excluded proxy respondents (n = 15,760), individuals with self-reported history of any cancer except non-melanoma skin cancer at baseline (n = 49,318), poor health based on self-rated health status (n = 8,365) or end-stage renal disease (n = 769) because of reduced life expectancy, individuals with any cancer except non-melanoma skin cancer as confirmed by cancer registry (n = 1,836), and those with cancer death but no report in cancer registry (n = 2,041). Additionally, we excluded respondents with energy intake outliers, defined as more than two interquartile ranges above the 75^th^ or below the 25^th^ percentile of sex-specific log-transformed intake (n = 4,290; 2,503 men and 1,787 women). The final study sample included 484,020 individuals (288,624 men and 195,396 women).

### Ascertainment of Cancer

Information about first primary incident colorectal cancer was obtained by linking the study participant’s identifiers with eight original state cancer registries and three additional states that participants tended to move during follow-up (Arizona, Nevada, and Texas). Previous validation study has shown this approach to be about 90% accurate [16]. The follow-up period for incident cancer was through December 31, 2006. Cases were defined as those who were diagnosed with first primary colorectal cancer. Anatomical site of colorectal cancer was identified based on the *International Classification of Disease for Oncology* (3rd edition); codes C180–C184: proximal colon, C185–C187: distal colon, C199 and C209: rectum, and C188–C189 and C260: not otherwise specified.

### Ascertainment of Diabetes

History of diabetes was assessed by self-report. In the baseline questionnaire, participants were asked if a doctor has told them that they have diabetes. Respondents who answered “yes” to this question were determined to have diabetes. This questionnaire, however, did not include any question about the type or onset of diabetes.

### Diet Quality

A validated self-administered questionnaire consisting of 124 food items was used to collect information about frequency and amount of dietary intake over the past 12 months [17]. We assessed diet quality by calculating scores for the Healthy Eating Index-2005 (HEI-2005), using data completed at study entry. The index, created by the National Cancer Institute and the U.S. Department of Agriculture, determines the concordance between one’s diet and *Dietary Guidelines for Americans, 2005* [18]. We chose HEI-2005 as the measure of diet quality because adherence to the dietary guidelines is the basis for healthy nutrition for general public as well as for the management of type 2 diabetes. In addition, the higher scores of HEI-2005 have been associated with the lower risk of diabetes [19]. The HEI-2005 score ranges from 0 to 100, with higher scores indicating greater concordance with the guidelines. The total score is the sum of scores for 12 components: six components with scores from 0 to 5 including total fruit, whole fruit, total vegetables, dark green/orange vegetables and legumes, total grains, whole grains; five components with scores from 0 to 10 including milk, meat and beans, oils, saturated fat, sodium; and one component with score from 0 to 20 including calories from solid fats, alcoholic beverages, and added sugars. The dietary data was linked with the MyPyramid Equivalent Database to calculate pyramid equivalents for the HEI-2005 components. The components scores, then, were calculated from the MyPyramid equivalents while adjusting for energy density (per 1000 kcal). We categorized the total HEI-2005 scores into quartiles (Q), with Q1 representing least concordance (poor-quality diet) and Q4 representing most concordance with dietary guidelines (good-quality diet).

### Statistical Analysis

All statistical analyses were performed using SAS 9.2 (SAS Institute, Cary, NC). We performed all analyses for men and women separately, to compare the results with previous reports, as some studies have shown different findings among men and women [4], [20], [21]. We then performed analyses for men and women combined, if the results were similar. We calculated incidence rates of colorectal cancer as the number of cases per 100,000 person-years of follow-up by history of diabetes and by quartiles of HEI-2005. For each participant, the duration of follow-up was calculated as the time between study entry until the diagnosis of colorectal cancer, moved outside registry area, death, or end of follow up period, whichever was earlier. We constructed age-adjusted Cox proportional hazard models to estimate the hazard ratio (HR) and corresponding 95% confidence intervals (CI) of incident colorectal cancer associated with history of diabetes at baseline. Models were adjusted for potential confounders, including demographic, clinical and dietary factors, as described below. For categorical confounders with missing data, we created a missing-data category, if the proportion of missing data was ≤5%. A sub-analysis was used to include the variables with missing data >5%.

To test the impact of diet quality in the association between diabetes and colorectal cancer, we estimated models with and without dietary factors. The initial model (Model 1), adjusted for the potential confounders based on previous studies [3], [4], [10]: age, sex (in combined men and women), race/ethnicity, education, body mass index (BMI), smoking, physical activity, replacement hormone therapy in women, family history of colon cancer, and vitamin and mineral supplements. Next, in the full model (Model 2), we further adjusted for diet quality (sex-specific HEI-2005 quartiles) and energy intake (kcal/day, continuous) to test if dietary factors confounded the association between diabetes and colorectal cancer.

To examine the combined effect of diet and diabetes, we first tested a two-way interaction between diabetes and HEI-2005 quartiles to evaluate whether the association between diabetes and colorectal cancer differed by categories of HEI-2005. Then, we created a new variable with 8 categories by combining diabetes status (2 categories) and HEI-2005 quartiles. We repeated the full model (Model 2), including this combined variable instead of its components. We also estimated the proportion of incident cases of colorectal cancer that would be prevented if all participants with diabetes had a good diet quality (HEI-2005 Q4). First, we multiplied the number of person-years in each diabetes/HEI-2005 category by the incident rate in the diabetes/good diet category. Next, we subtracted the calculated incidence from the actual incidence to get the number of cases that would be prevented; the numbers were presented as the percent of the total incident cases among individuals with diabetes.

Finally, we estimated the HR and corresponding 95% CI for incident colorectal cancer by each anatomical sub-site: colon, including proximal and distal colon, and rectum.

#### Sensitivity analyses

Because cancers early in the follow-up might be related to the diet before study entry, we repeated the analysis after excluding incident cases during the first 2 years of follow-up [22]. In addition, because data on two potential confounders were available only for a subgroup who completed the risk factor questionnaire (n = 334,908), we further adjusted for these two variables, including regular use of non-steroidal anti-inflammatory drugs (NSAIDs), i.e., at least 3 times/week, and history of screening for colorectal cancer, i.e., fecal occult blood test, sigmoidoscopy, proctoscopy or colonoscopy.

## Results

At baseline, individuals with diabetes were on average older and less likely to be White, college graduate, physically active, use multivitamin regularly, and have family history of colon cancer than those without diabetes. In addition, individuals with diabetes were more likely to be obese (BMI ≥30), have never used replacement hormone (for women), and use NSAIDs regularly (Table 1). In addition, individuals with diabetes had a more favorable dietary score than those without diabetes. Further, men with diabetes were less likely and women with diabetes were more likely to have never smoked.

**Table 1:** Baseline Characteristics of the Study Sample.

	Men		Women	
	No diabetesn = 260,680	Diabetesn = 27,944	No diabetesn = 181,895	Diabetesn = 13,501
**Demographic**				
Age, years, mean	62.0	63.0	61.8	62.7
Race/ethnicity (% White)	93.0	88.4	90.1	80.9
Education (% college graduate)	45.7	36.7	30.7	21.0
**Clinical**				
Physical activity (% ≥5 times/week)	21.8	18.8	16.6	12.0
Smoking (% never)	29.9	24.5	44.1	45.2
Body mass index (% BMI <30 kg/m^2^)	79.2	62.4	76.9	46.6
Multivitamin use (% >6 times/week)	40.7	40.0	47.1	42.9
Family history of colon cancer (%)	8.3	7.9	9.5	8.8
Replacement hormone use (% never)^b^	–	–	45.5	60.3
NSAIDs use (% regular)^a^	45.9	55.0	39.0	49.4
Screening for colorectal cancer (%)^a^	72.8	75.4	60.3	59.6
**Diet**				
Calories, mean	2018.6	1953.0	1566.0	1593.7
Healthy Eating Index-2005, mean				
- Total score (0–100)	65.1	68.7	69.5	71.8
- Total fruit (0–5)	4.1	4.2	4.4	4.4
- Whole fruit (not juice) (0–5)	4.0	4.3	4.4	4.5
- Total vegetables (0–5)	4.0	4.1	4.3	4.3
- Dark green & orange vegetables & legumes (0–5)	2.4	2.5	3.1	3.1
- Total grains (0–5)	4.2	4.4	4.3	4.4
- Whole grains (0–5)	1.8	2.1	1.9	2.1
- Milk (0–10)	5.0	5.5	5.5	5.8
- Meat and beans (0–10)	9.0	9.3	8.5	9.0
- Oils (0–10)	6.6	7.0	6.9	7.3
- Saturated fat (0–10)	7.4	6.9	7.6	7.2
- Sodium (0–10)	4.4	3.0	4.3	3.2
- Calories from solid fats, alcoholic beverages & addedsugars (0–20)	12.1	15.3	14.4	16.5

a Data from risk factor questionnaire (n = 16,641 are missing);

b % are among women.

### Diabetes-colorectal Cancer Association: Impact of Diet

During an average 9.2 years of follow-up (median: 10.5 years, range: 0.003–11.2 years), we identified 7,598 (5,130 men and 2,468 women) new cases of first primary colorectal cancer. Of these, 872 cases had a history of diabetes at baseline. Among both men and women, the age-adjusted models showed that both history of diabetes and poor diet were associated with increased risk of colorectal cancer (Table 2). After controlling for non-dietary risk factors (Model 1), the association between diabetes and colorectal cancer was attenuated, yet remained significant both in men and in women, suggesting that diabetes was associated with an increased risk of colorectal cancer independent of non-dietary confounders. Further adjustment for HEI-2005 and energy intake (Model 2) did not attenuate the estimates, suggesting that diet quality was not a confounder in the association between diabetes and colorectal cancer above and beyond non-dietary confounders. In addition, an increased risk of colorectal cancer was associated with obesity in men (HR: 1.18, 95% CI: 1.11, 1.26), but not in women (HR: 1.06, 95% CI: 0.96, 1.17).

**Table 2:** Diabetes, Diet Quality and Risk of Colorectal Cancer.

	Cases/100,000person-years	Age-adjusted	Model 1^a^	Model 2^b^
**Men and Women**				
Diabetes				
Yes	230	1.41 (1.31, 1.51)	1.27 (1.18, 1.36)	1.32 (1.23, 1.42)
No	156	1.00	1.00	1.00
HEI-2005 quartiles, diet quality				
Quartile 1, poor diet	195	1.50 (1.41, 1.60)	–	1.35 (1.26, 1.44)
Quartile 2	166	1.24 (1.16, 1.32)	–	1.17 (1.09, 1.25)
Quartile 3	145	1.07 (1.00, 1.14)	–	1.04 (0.97, 1.11)
Quartile 4, good diet	140	1.00	–	1.00
**Men**				
Diabetes				
Yes	248	1.33 (1.22, 1.44)	1.23 (1.13, 1.34)	1.29 (1.18, 1.40)
No	178	1.00	1.00	1.00
HEI-2005 quartiles, diet quality				
Quartile 1, poor diet	227	1.53 (1.42, 1.65)	–	1.37 (1.26, 1.49)
Quartile 2	186	1.22 (1.12, 1.32)	–	1.14 (1.05, 1.24)
Quartile 3	167	1.08 (0.99, 1.17)	–	1.05 (0.96, 1.14)
Quartile 4, good diet	159	1.00	–	1.00
**Women**				
Diabetes				
Yes	195	1.50 (1.31, 1.71)	1.36 (1.19, 1.56)	1.41 (1.23, 1.61)
No	124	1.00	1.00	1.00
HEI-2005 quartiles, diet quality				
Quartile 1, poor diet	149	1.44 (1.29, 1.61)	–	1.30 (1.15, 1.46)
Quartile 2	138	1.28 (1.15, 1.44)	–	1.22 (1.09, 1.37)
Quartile 3	114	1.05 (0.93, 1.18)	–	1.02 (0.91, 1.15)
Quartile 4, good diet	113	1.00	–	1.00

a adjusted for age (continuous), sex (in combined men and women), race/ethnicity (non-Hispanic White; non-Hispanic Black; Hispanic; other), education (≤ high school; post high school graduate; some college; ≥ college graduate), body mass index (BMI) (<30, ≥30), smoking (never; past, ≤20/day; past, >20/day; current, ≤20/day; current, >20/day), physical activity (never/rarely; 1–3 times/month; 1–2 times/week; 3–4 times/week; ≥5 times/week), replacement hormone therapy in women (never; currently; formerly), family history of colon cancer (yes; no), and vitamin and mineral supplements (non; <1 time/week; 1–3 times/week; 4–6 times/week; >6 times/week).

b Model 1+ Diet quality score quartiles+energy (kcal/day).

In the sensitivity analysis, when we repeated the analysis after excluding those cases that were diagnosed during the first two years of follow-up (995 men and 418 women) the results were unchanged. Specifically, HR of colorectal cancer associated with diabetes was 1.30 (95% CI: 1.20, 1.41, for men and women combined).

In addition, results of the sensitivity analysis showed that including two more confounders, i.e., use of NSAIDs and history of screening for colorectal cancer, to the multivariate model did not affect the extent of the association between colorectal cancer and either diabetes or diet quality (Diabetes, HR: 1.35, 95% CI: 1.23, 1.48; HEI-2005 Q1 vs. Q4, HR: 1.32, 95% CI: 1.21, 1.44, for men and women combined).

### Combined Effect of Diet and Diabetes

**Figure 1:**
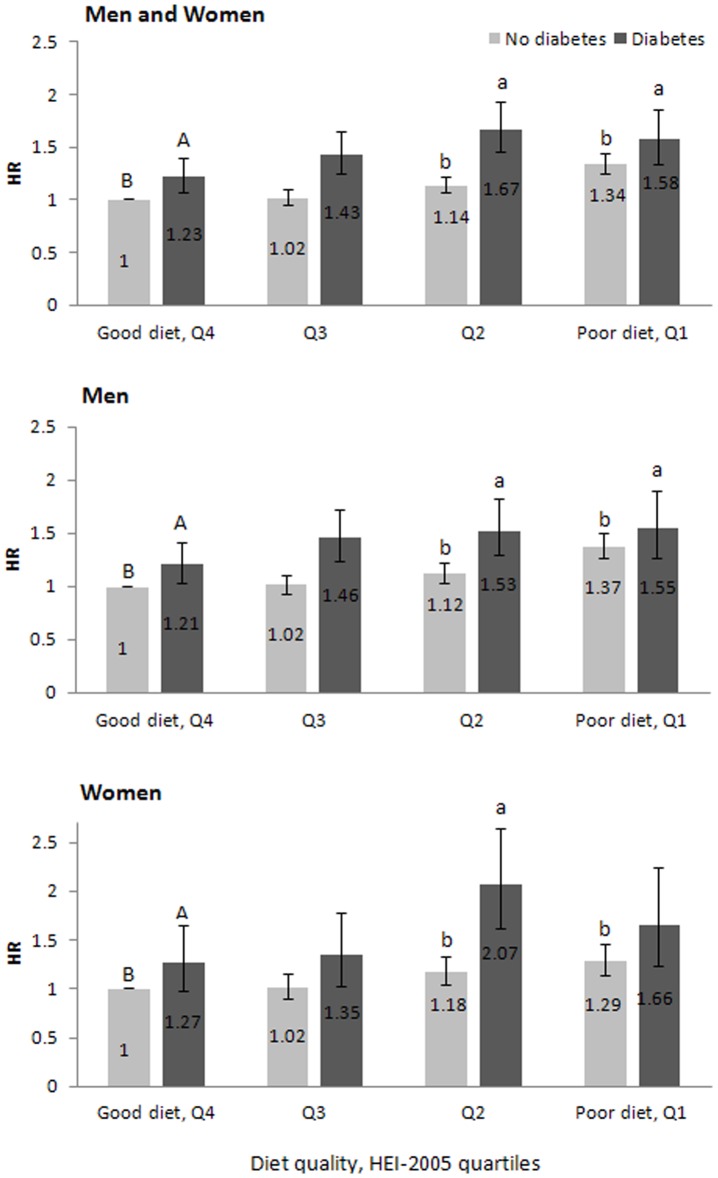
Combined Effect of Diet Quality and Diabetes on Risk of Colorectal Cancer among Men and Women Combined (Upper Panel), Men (Middle Panel), and Women (Lower Panel). Error bars indicate 95% confidence interval. In each panel, columns with letter ‘a’ exhibit a significantly greater HR compared to the column with letter ‘A’ (Diabetes and Good diet) and columns with letter ‘b’ exhibit a significantly greater HR compared to the column with letter ‘B’ (No diabetes and Good diet) (P<0.05).

Examination of the combined effect of diet and diabetes suggests that diabetes and poor diet exert an additive, rather than a synergistic, effect on the risk of colorectal cancer in both men and women. The association between diabetes and colorectal cancer did not vary by categories of HEI-2005, i.e., there was no interaction between diabetes and diet quality (P_Interaction = _0.2). In addition, among all participants with a good diet (HEI-2005 Q4), history of diabetes was associated with a HR of 1.23 (95% CI: 1.07, 1.40, P = 0.003) compared with no diabetes and good diet. Having both diabetes and poor diet (HEI-2005 Q1) increased the risk of colorectal cancer with a HR of 1.58 (95% CI: 1.34, 1.86, P<0.0001) (Fig. 1). When we used diabetes and good diet, as the reference group, the risk of colorectal cancer increased for lower quality diets among men (HEI-2005 Q1, P = 0.04; Q2, P = 0.03) and women (HEI-2005 Q2, P = 0.004) with diabetes, suggesting that having a poor diet posed an additional risk to people with diabetes. Moreover, we estimate that of the 872 incident colorectal cancers in individuals with diabetes, 16% (138 cases) might have been prevented, if all participants with diabetes had a good diet.

### Site-specific Associations

Diabetes increased the risk of overall and site-specific colorectal cancer in a model including dietary and non-dietary confounders, among both men and women (Table 3). Diabetes was more strongly associated with proximal than distal colon cancer (e.g., HR: 1.33 vs. HR: 1.20, in men and women combined), while poor diet was associated with a weaker risk of proximal than distal colon cancer (e.g., HR: 1.18 vs. HR: 1.46, in men and women combined). In addition, poor diet was associated with a stronger risk of rectum than colon cancer in men (HR: 1.64 vs. HR: 1.29), but not in women (HR: 1.25 vs. HR: 1.29).

**Table 3:** Diabetes, Diet Quality and Risk of Colorectal Cancer by Sub-site.

	Colon	Proximal colon	Distal colon	Rectum
**Men and Women**				
No. of cases	5292	3063	2229	2061
Diabetes				
Yes	1.27 (1.17, 1.39)	1.33 (1.19, 1.49)	1.20 (1.04, 1.37)	1.36 (1.18, 1.56)
No	1.00	1.00	1.00	1.00
HEI-2005 quartiles, diet quality				
Quartile 1, poor diet	1.29 (1.19, 1.40)	1.18 (1.07, 1.31)	1.46 (1.28, 1.65)	1.51 (1.32, 1.72)
Quartile 2	1.11 (1.02, 1.20)	1.05 (0.94, 1.16)	1.21 (1.07, 1.37)	1.30 (1.14, 1.48)
Quartile 3	1.00 (0.93, 1.09)	0.95 (0.85, 1.05)	1.10 (0.97, 1.25)	1.13 (0.99, 1.30)
Quartile 4, good diet	1.00	1.00	1.00	1.00
**Men**				
No. of cases	3508	1945	1563	1440
Diabetes				
Yes	1.24 (1.12, 1.38)	1.30 (1.13, 1.49)	1.17 (0.99, 1.37)	1.34 (1.14, 1.57)
No	1.00	1.00	1.00	1.00
HEI-2005 quartiles, diet quality				
Quartile 1, poor diet	1.29 (1.17, 1.42)	1.17 (1.02, 1.33)	1.47 (1.26, 1.71)	1.64 (1.40, 1.93)
Quartile 2	1.04 (0.95, 1.15)	0.97 (0.86, 1.11)	1.15 (0.99, 1.34)	1.38 (1.18, 1.62)
Quartile 3	0.98 (0.89, 1.08)	0.90 (0.79, 1.03)	1.09 (0.94, 1.27)	1.23 (1.05, 1.45)
Quartile 4, good diet	1.00	1.00	1.00	1.00
**Women**				
No. of cases	1784	1118	666	621
Diabetes				
Yes	1.37 (1.16, 1.60)	1.41 (1.15, 1.72)	1.29 (0.99, 1.69)	1.43 (1.08, 1.88)
No	1.00	1.00	1.00	1.00
HEI-2005 quartiles, diet quality				
Quartile 1, poor diet	1.29 (1.12, 1.48)	1.21 (1.02, 1.45)	1.43 (1.14, 1.81)	1.25 (0.99, 1.58)
Quartile 2	1.24 (1.09, 1.42)	1.18 (1.00, 1.40)	1.37 (1.09, 1.72)	1.13 (0.90, 1.42)
Quartile 3	1.06 (0.92, 1.22)	1.03 (0.87, 1.22)	1.12 (0.89, 1.42)	0.95 (0.75, 1.20)
Quartile 4, good diet	1.00	1.00	1.00	1.00

## Discussion

In this large prospective study, we examined the role of diet quality on the association between type 2 diabetes and colorectal cancer. Our findings replicated previous reports in showing that men and women with a history of diabetes have a moderately increased risk of colorectal cancer compared with those without diabetes, after adjusting for potential confounders. In addition, the HEI-2005 scores at baseline did not account for the excess risk of incident colorectal cancer associated with diabetes. A history of both diabetes and poor diet quality (HEI-2005 Q1) further increased the risk of colorectal cancer. Our findings suggest that diabetes-specific risk was greater for proximal colon cancer, while diet quality-specific risk was greater for distal colon cancer.

Our results infer that diabetes and poor diet quality impact the risk of colorectal cancer independently and additively. Consistent with three recent meta-analyses [7], [23], [24], we found that diabetes is associated with a modest increase of colorectal cancer risk both in men and women. However, HEI-2005 was not a confounding factor, despite the fact that poor diet is a shared risk factor for both type 2 diabetes [19] and colorectal cancer [10]. Although previous studies have controlled for one or more dietary factors, to our knowledge no studies have examined whether diet confounds the association between diabetes and colorectal cancer. Analysis of 15 years of follow-up data from the Cancer Prevention Study (CPS) II Nutrition Cohort, suggested that dietary factors, considered together with several socioeconomic variables did not affect the findings [20], although the impact of dietary factors alone was not reported.

The general presumption that a poor diet might account in part for the association between diabetes and colorectal cancer is predicated on the expectation that people with type 2 diabetes consume a less prudent diet than those without diabetes. However, the results of the HEI-2005 score indicated a more favorable diet among individuals with diabetes compared to those without diabetes. Consistent with this finding, the results from the first Cancer Prevention Study of 1.2 million participants indicated that individuals with diabetes were more likely to eat fresh fruit, cooked vegetables, green salad, meat/poultry, and milk and less likely to consume fried foods and alcohol than those without diabetes [4]. Considering the long lag between exposure to potential dietary risk factors and development of colorectal cancer, it might be argued that the increased incidence of colorectal cancer in diabetes is associated with dietary modifiers perhaps years before the diagnosis of diabetes. Although we cannot formally exclude this possibility, our finding of similar results after excluding the cancers that occurred during the first two years of follow-up makes this possibility less likely. In addition, we did not have data regarding duration of diabetes and diet quality before diagnosis of diabetes. Given that poor diet is a risk factor for diabetes [6], [19], an improvement in diet after diagnosis of diabetes may potentially explain why diet did not play a role in the association between diabetes and colorectal cancer.

Our findings further indicate that the combined effect of diet and diabetes was additive. People with diabetes and a favorable diet had about 20% greater risk of colorectal cancer compared to those without diabetes and with a good diet. However, having a less favorable diet, in addition to diabetes, increased the risk of colorectal cancer up to about 60%. This observation raises the clinical implication that improving diet quality in type 2 diabetes might lower the risk of colorectal cancer. In addition, while attention is usually focused on the confounding effect of diet as a shared risk factor for colorectal cancer, our results suggest that the association between diabetes and colorectal cancer is independent from known confounding factors; there are as yet unknown factors that further predispose diabetics to the development of colorectal cancer.

Our findings also suggest that diabetes increases the risk of cancer in the proximal colon more than that of the distal colon. This finding may have important clinical implication, since strategies for screening colorectal cancer might differ in individuals with high versus low risk for proximal colon cancer [25]. Similar to our findings, four cohort studies have shown that a history of diabetes was associated with a greater risk of proximal than distal colon cancer in women [3], [14] and men [8], [26]. However, a case-control study in men and women [13] showed an association between diabetes and colon cancer only for distal colon. In addition, most previous work has failed to find an association between diabetes and rectal cancer, especially among women [24], which might be because of the small number of cases. Our findings, drawn from a large cohort with 5,292 incident cases of colon cancer and 2,061 of rectal cancer, suggest that diabetes is associated with an increased risk of both colon and rectal cancer, consistent with two meta-analyses [7], [23].

Regarding site-specific association for diet, our findings are in agreement with most, but not all [27], [28], studies indicating that diet-related risk of colon cancer is site-specific, with stronger risk for distal than for proximal colon cancer. Previous work on the NIH-AARP population reported an inverse association between diet quality, quintile 5 vs. quintile 1 of HEI-2005, and the risk of distal colon cancer both in men and in women [10]. In addition, prospective studies that examined food groups have shown that lower intake of milk [29], and fruits and vegetables [30], and higher intake of red and processed meat [31] were associated with higher risk of distal colon cancer. However, a case-control study suggests an association between fat and proximal colon cancer, possibly because of the interaction with bile mechanism [32].

In addition, our data showed that the association between diabetes and colorectal cancer was independent of main confounders including obesity. Data suggest that the effect of obesity on risk of colorectal cancer is direct and independent of diet and physical activity [33]. Similar to previous studies [34], [35], we found a stronger association between obesity and colorectal cancer in men than women. The reason for this sex difference may be because in our sample women were mainly postmenopausal, as the association between obesity and colorectal cancer is weaker in postmenopausal than premenopausal women [36].

Several biologically plausible mechanisms have been described in the association between diabetes and colorectal cancer. One major mechanism is based on the systemic manifestation of insulin resistance in type 2 diabetes, i.e. hyperinsulinemia [37], [38]. Insulin stimulates the proliferation of cells in several organs, mainly through activation of insulin-like growth factor-1 receptor. In addition, high level of plasma glucose may contribute to colorectal cancer risk by providing glucose as the only energy source for tumor cells [39]. Other potential mechanisms involve changes in luminal factors, such as prolonged bowel transit time [40], changes in bile acid metabolism [41], compositional change in gut microbiota [42], and decrease in gut mucosal layer [43]. Given our finding of a site-specific association between diabetes and colorectal cancer, it appears that a combination of systemic disturbances and luminal factors, rather than systemic factors alone, may better explain the association between diabetes and colorectal cancer. Future research should investigate diabetes-related luminal abnormalities with a focus on any possible differential effect of diabetes on proximal than distal colon.

This study has potential limitations. First, history of diabetes was self-reported. Thus, individuals with subclinical diabetes were categorized as not having diabetes, and so the association between diabetes and colorectal cancer may have underestimated. Second, we did not have data on several aspects of diabetes that may potentially influence risk of colorectal cancer, including diabetes duration [3], anti-diabetic medications including insulin [44]. In addition, we could not distinguish between type 1 and type 2 diabetes. However, since type 2 diabetes accounts for more than 90% of the cases of diabetes in adults [2], our results likely apply to type 2 diabetes. Third, we did not identify the new cases of diabetes after the baseline survey, and so the incident diabetes cases were categorized as non-diabetes. This misclassification may have attenuated our results, but only very minimal because of the large ratio of non-diabetes to diabetes population in our study (∼10∶1). The strengths of our study are the large sample size, availability of data on both dietary and non-dietary risk factors of colorectal cancer, the registry diagnosis of cancer and the long duration of follow-up.

In conclusion, a history of diabetes was associated with a moderate risk of colorectal cancer in men and women. Diabetes and poor diet (measured by HEI-2005) were two independent and additive risk factors for colorectal cancer. Improving diet quality may substantially improve, although not eliminate, the risk of colorectal cancer among people with type 2 diabetes. In addition, the association between type 2 diabetes and proximal colon cancer may have clinical implications for screening strategies among these people.
